# Supplementary material for: Reversible conversion between skyrmions and skyrmioniums
Source: Nat Commun. 2023 Jun 9;14:3406. doi: 10.1038/s41467-023-39007-1 (PMC10256730; doi:10.1038/s41467-023-39007-1)
Supplement: Supplementary file 1 — Supplementary Information [file 41467_2023_39007_MOESM1_ESM.pdf]

Supplementary information of "Reversible conversion between skyrmions and skyrmioniums"

Sheng Yang, [#,1] Yuelei Zhao, [#,1] Kai Wu,[1] Zhiqin Chu,[2,3] Xiaohong Xu,[4,5] Xiaoguang Li, [*,6] Johan Åkerman[*,7,8,9] and Yan Zhou[*,1]

[1]School of Science and Engineering, The Chinese University of Hong Kong, Shenzhen, 518172, China.

[2] Department of Electrical and Electronic Engineering, The University of Hong Kong, Hong Kong, 999077, China

[3]School of Biomedical Sciences, The University of Hong Kong, Hong Kong, 999077, China

[4]Research Institute of Materials Science of Shanxi Normal University & Collaborative Innovation Center for Shanxi Advanced Permanent Magnetic Materials and Technology, Linfen, 041004, China.

[5]School of Chemistry and Materials Science of Shanxi Normal University & Key Laboratory of Magnetic Molecules and Magnetic Information Materials of Ministry of Education, Linfen, 041004, China.

[6]Shenzhen Key Laboratory of Ultraintense Laser and Advanced Material Technology, Center for Advanced Material Diagnostic Technology, and College of Engineering Physics, Shenzhen Technology University, Shenzhen, 518118, China

[7]Department of Physics, University of Gothenburg, Gothenburg, 41296, Sweden.

[8]Center for Science and Innovation in Spintronics, Tohoku University, 2-1-1 Katahira, Aoba-ku, Sendai 980-8577 Japan

[9]Research Institute of Electrical Communication, Tohoku University, 2-1-1 Katahira, Aoba-ku, Sendai 980-8577 Japan.

#These authors contributed equally.

Corresponding Authors

* E-mail: lixiaoguang@sztu.edu.cn

* E-mail: johan.akerman@physics.gu.se

* E-mail: zhouyan@cuhk.edu.cn

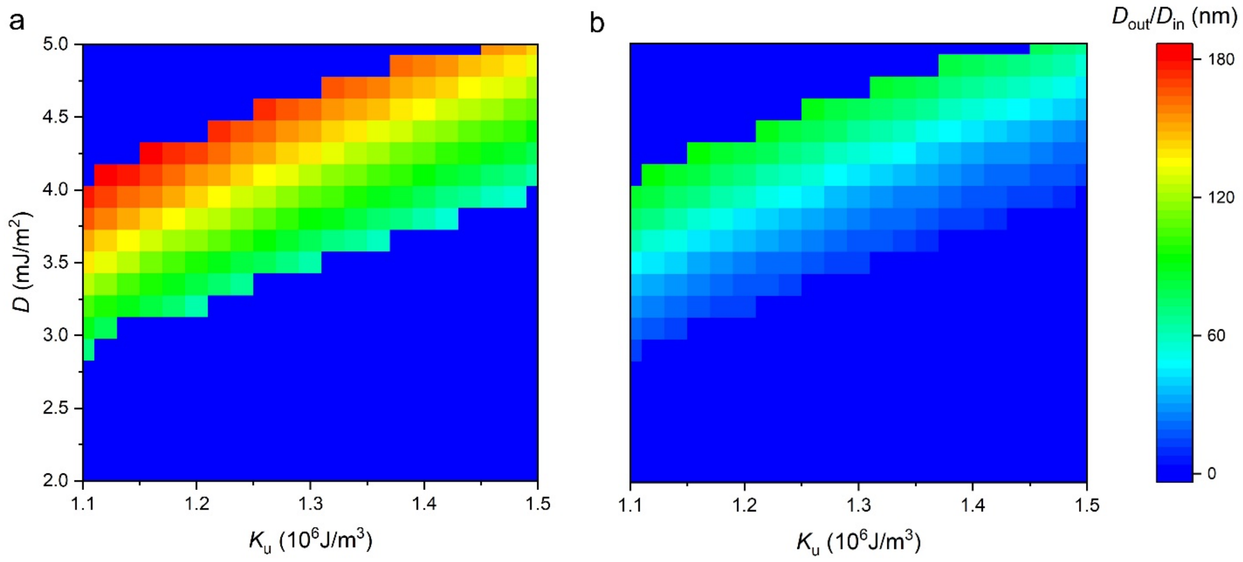

Supplementary Figure 1. **a**, Diagram of the outer diameter D_{out}. **b**, the inner diameter D_{in} of the skyrmionium as a function of DMI constant D and the magnetic anisotropy energy density K_u.

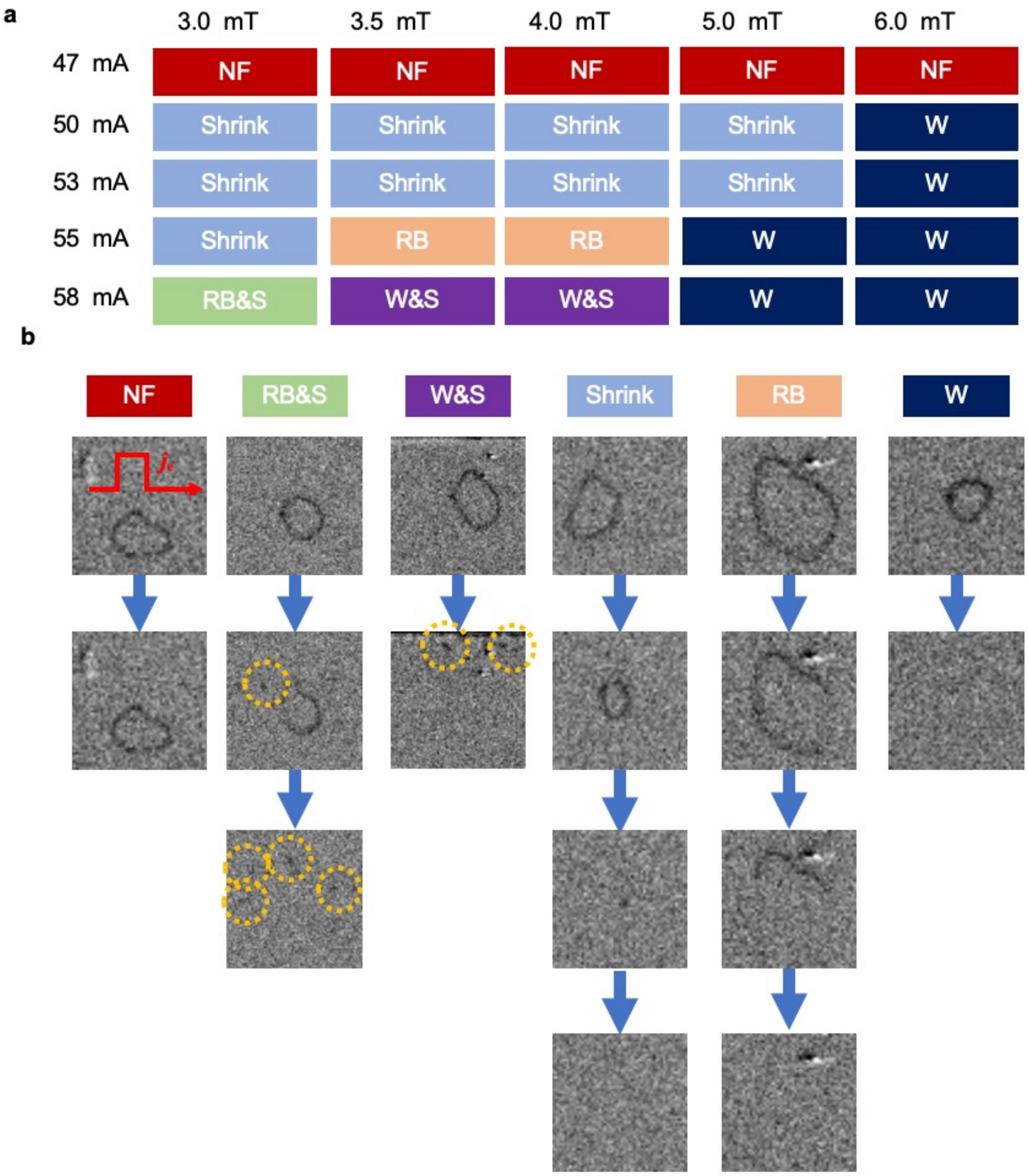

Supplementary Figure 2. NF (No Function): the skyrmionium does not change significantly under the stimulation of pulses. Shrink: the skyrmionium shrinks and simultaneously moves without any SkHE. RB (Ring breaks): the skyrmionium ring breaks. RB&S (Ring break and skyrmion): the skyrmionium ring breaks, and multiple skyrmions are created. W&S (Wiped and skyrmion): the skyrmionium is obliterated, and multiple skyrmions are created. W (Wipe): the skyrmionium is obliterated.

The results of the current-driven skyrmionium dynamics in our device are shown in Fig. S2b. We use multiple square pulses applied in the device to drive the skyrmionium into motion. Unless otherwise stated, all square pulse and sinusoidal pulses used in the experiments have a pulse width of 50 μs. The skyrmionium is quite sensitive to out-of-plane field and current amplitude, as shown in Fig. S2a. When the square pulses are at amplitude $I_{sq} \leq 47$ mA (1.68×10^{11} A/m^2), the skyrmionium does not change significantly. We have marked the box with NF for 'No Function' in Fig. S2a and Fig S2b. Multiple 47 mA square pulses can generate enormous amounts of heat, but the skyrmionium can remain stationary, implying that heat cannot drive the skyrmionium into motion or change its size. As we gradually increase the I_{sq}, we soon find that 50 mA (1.79×10^{11} A/m^2) is the threshold I_{sq} above which the skyrmionium is driven into motion.

When the magnetic field is set to be 3.0 mT and the square pulse amplitudes I_{sq} are varied from 50 mA to 55 mA (1.79×10^{11} A/m^2 to 2.00×10^{11} A/m^2), we observe that the skyrmionium shrinks and simultaneously moves without SkHE (see Fig. S2b). These moving and shrinking processes have already been discussed in detail in the research article. However, when I_{sq} increases to 58 mA (2.01×10^{11} A/m^2), the skyrmionium undergoes a ring break process, and the device generates some skyrmions at the same time (labelled as RB&S, see Fig. S2b). Specifically, the device can intrinsically generate skyrmions using square pulses with $I_{sq} = 58$ mA under a 3.0 mT to 4.0 mT +z field. Please refer to Supplementary Video 7 for more details. As the skyrmions are created in a different location from that of the skyrmionium, they are very unlikely to be the fragments of the skyrmionium. Therefore, the creation of the skyrmions is irrelevant to the destruction process of the skyrmionium.

When the magnetic field is increased to 4.0 mT, we can observe the same moving and shrinking process when $I_{sq} \leq 55$ mA, and we can also observe the ring breaking process (labelled as RB, see Fig. 2b). When I_{sq} is increased to 58 mA, the skyrmionium is directly obliterated, and multiple skyrmions are created (labelled as W&S, see Fig. S2b). Again, the creation of these skyrmions is irrelevant to the destruction of the skyrmionium. It is worth noting that the ring breaking process can only occur in a narrow range (field of 3.5 mT to 4 mT, $I_{sq} = 55$ mA or field of 3.0 mT, $I_{sq} = 58$ mA). Theoretically, the two skyrmions with opposite Q form the skyrmionium ring, and the process of ring breaking occurs because the inner skyrmion experiences the opposite SkHE to the outer skyrmion[1]. Once SOT is applied to the skyrmionium, one side of the skyrmionium ring becomes thin and can be easily broken.

As the range increase from 3.5 mT to 4 mT, the same moving and shrinking process occurs when $I_{sq} \leq 55$ mA. However, the skyrmionium can be obliterated directly when $I_{sq} \geq 55$ mA (labelled as W, see Fig. S2b). With a 6.0 mT field, the skyrmionium can be obliterated directly as long as $I_{sq} \geq 50$ mA.

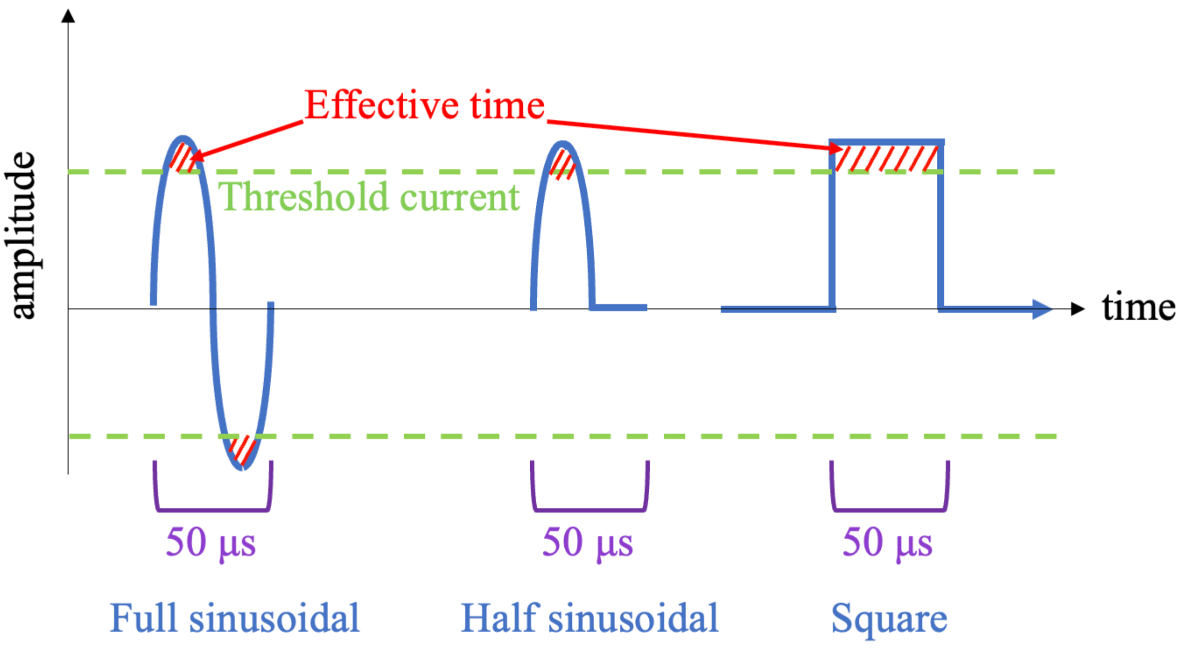

Supplementary Figure 3. The configurations of different waveforms.

Fig.1c shows two ways of producing skyrmionium: via a square pulse and a sinusoidal pulse. As presented in Fig. 2 and 3, the square pulse was used to transform skyrmion bag into skyrmionium under an external field of 3 mT, while the sinusoidal pulse was utilized to break down stripe domain to create skyrmionium under an external field of 6 mT. External field plays a crucial role in skyrmion bag's motion or deterioration. A Supplementary video 8 is added to explain why sinusoidal pulse was used in decomposing the net domain or the skyrmion bag. In the first part of the video, the skyrmion expands gradually under an external field of 6 mT with sinusoidal pulses applied (either full sinusoidal pulse or half sinusoidal pulse); in contrast, square pulse instantaneously switches the magnetization, leaving behind only a few stripe domains. To generate skyrmionium, the net domain needs to be created in the first place, and then transformed into skyrmion bag. Compared with the square pulse, this process can be

progressively controlled by the sinusoidal pulse as due to its short effective time (during which the current amplitude is higher than the threshold to drive magnetic structures into motion) as shown in Fig. S3. The second part of video further demonstrates that, while the full and half sinusoidal pulses break down the net domain gradually, the effect of square pulses is uncontrollable even with a single step. The inherent feature of the square pulse that leads to this behavior is its longer effective time.

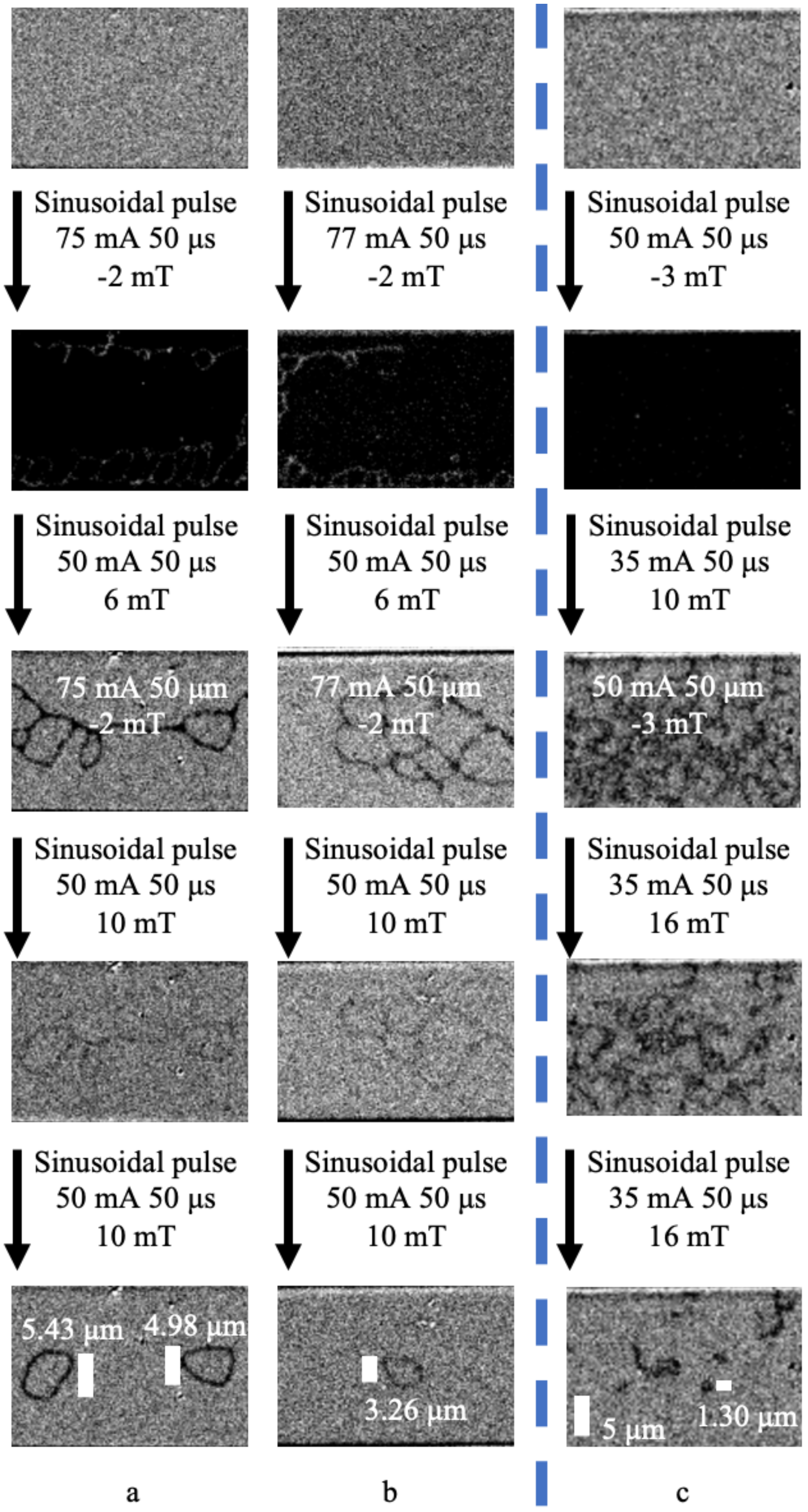
Sinusoidal pulse
75 mA 50 µs
-2 mT
Sinusoidal pulse
77 mA 50 µs
-2 mT
Sinusoidal pulse
50 mA 50 µs
-3 mT
Sinusoidal pulse
50 mA 50 µs
6 mT
Sinusoidal pulse
50 mA 50 µs
6 mT
Sinusoidal pulse
35 mA 50 µs
10 mT
75 mA 50 µm
-2 mT
77 mA 50 µm
-2 mT
50 mA 50 µm
-3 mT
Sinusoidal pulse
50 mA 50 µs
10 mT
Sinusoidal pulse
50 mA 50 µs
10 mT
Sinusoidal pulse
35 mA 50 µs
16 mT
Sinusoidal pulse
50 mA 50 µs
10 mT
Sinusoidal pulse
50 mA 50 µs
10 mT
Sinusoidal pulse
35 mA 50 µs
16 mT
5.43 µm
4.98 µm
3.26 µm
5 µm
1.30 µm
a
b
c

Supplementary Figure 4. **a-b** Skyrmioniums creation in Ta(3)/[Pt(0.5)/Co(0.5)]$_3$/Ru(2)/[Co(0.5)/ Pt(0.5)]$_3$/Ta(3). **c** Skyrmionium creation in Ta(3)/[Pt(0.5)/Co(0.6)]$_3$/Ru(2)/[Co(0.6)/ Pt(0.5)]$_3$/Ta(3). The skyrmionium has a relatively small diameter of about 1.30 μm.

The size of the skyrmionium in our sample (Ta(3)/[Pt(0.5)/Co(0.5)]$_3$/Ru(2)/[Co(0.5)/ Pt(0.5)]$_3$/Ta(3) was randomly distributed, yet we managed to slightly control its dimensions using certain methods. As we mentioned in the main text, the nucleation of skyrmions is premised to the creation of skyrmionium. As shown in Fig. S4a, we use sinusoidal pulse (75 mA, 50 μs) to generate skyrmions with a lower density, and use sinusoidal pulse (77 mA, 50 μs) to generate skyrmions with a higher density. Higher density of skyrmions can create a net domain with denser "holes", since the "holes" of the net domain are actually expanded by skyrmions, which means the size of the holes is smaller. As a result, the skyrmionium has a smaller size. The net domain in Fig. S4b had a higher density. As a result, the skyrmionium in Figure S4b is compressed and has a smaller size than shown in Fig. S4a (5.43 μm and 4.98 μm in Fig.S4a, and 3.26 μm in Fig.S4b). In Fig. S4c, we modified the stack structure to Ta(3)/[Pt(0.5)/Co(0.6)]$_3$/Ru(2)/[Co(0.6)/Pt(0.5)]$_3$/Ta(3), with the thickness of the Co layer increased. By applying the same methods, the size of the skyrmionium was reduced to 1.30 μm, which was closed to the imaging limitation of MOKE, which makes the current driven dynamics hard to be observed. Supplementary video 9 is uploaded to show the full creation process of the 1.30 μm skyrmionium (11 s of the video).

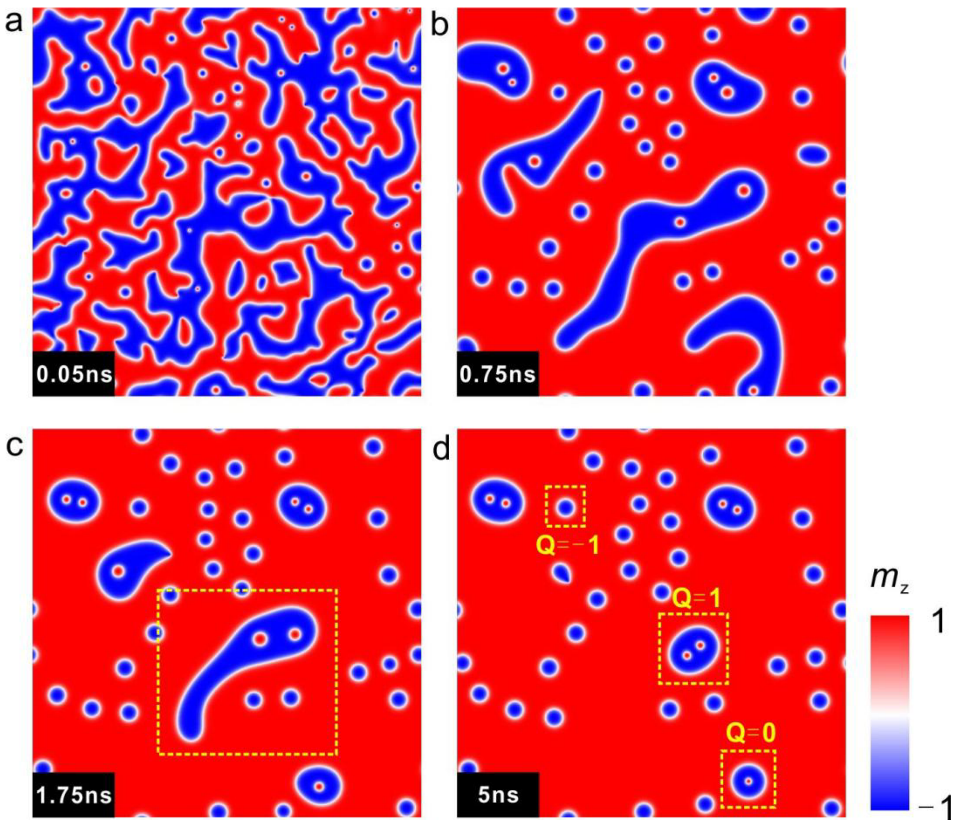

Supplementary Figure 5. a-d Simulated magnetization evolution of the magnetic thin film relaxed from a random initial state.

The diversity of skyrmion composites in a chiral magnet has been theoretically predicted [2-4]. In particular, the skyrmion solutions with arbitrary integer topological number can be stabilized in a conventional micromagnetic model with Dzyaloshinskii Moriya interaction (DMI)[2]. These cluster-like structures have been recently observed in liquid crystal [4] and exfoliated flakes of FeGeTe[6], and in general known as skyrmion bags or skyrmion sacks. Here we perform complementary micromagnetic simulation to demonstrate the nucleation process of the skyrmion bags with Q = 1. We adopted the same parameters as our previous simulations (c.f. *Computational Method*), and set the

DMI constant D=3.5mJ/m^2, the perpendicular external field $\mu_0 H_\perp$=10 mT. The simulated system is relaxed from an initial state with random magnetization orientations for 10ns, and Fig. S5 shows the snap shots of the out-of-plane component of the magnetization m_z at different time intervals. We observed that the magnetic domains with $m_z < 0$ quickly shrink to particle-like structures to minimize the Zeeman energy. Notably, at the time of 1.75ns, as shown in Fig. S5 c, the simulated transient state well reproduces the magnetic structures we observed in experiments (c.f. Fig. 3 e in the main text). The system is fully relaxed at a time of about 5 ns, and we observed the coexistence of skyrmion (Q = -1), skyrmionium (Q = 0) and skyrmion bag (Q = 1), as shown in Fig. S5 d. The above simulations demonstrate the possible stabilization of skyrmion bags in ferromagnetic thin films with interfacial DMI, e. g. the Pt/Co multilayers under investigation.

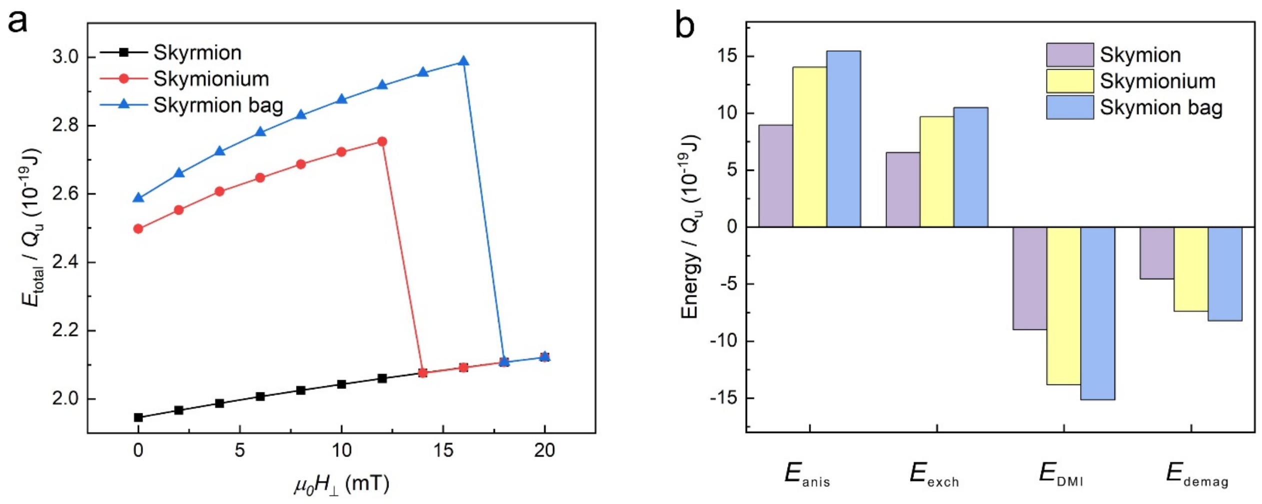

Supplementary Figure 6. Energy cost per topological charge of skyrmion, skyrmionium and skyrmion bag. **a** Total energy as a function of the perpendicular external field $\mu_0 H_\perp$. **b** Energy composition of the observed magnetic structures, including the energy

contributions from the magnetic anisotropy E_{anis}, Heisenberg exchange E_{exch}, Dzyaloshinskii–Moriya interaction E_{dmi}, and demagnetizing field E_{demag}.

We further analyze the energy states of skyrmion, skyrmionium and skyrmion bag to demonstrate the mechanism behind their stabilizations. For this purpose, it is reasonable in comparing, e.g., the energy of a skyrmionium and the energy sum of a pair of isolated skyrmions with $Q = \pm 1$ to demonstrate their relative stability. Since the energy of skyrmion with opposite Q degenerates, we can equally calculate the unfolded topological number $Q_u = \frac{1}{4\pi}\int \left|\boldsymbol{m}\cdot\left(\partial_x \boldsymbol{m} \times \partial_y \boldsymbol{m}\right)\right| dxdy$, and compare the free energy cost per topological charge E_{total}/Q_u. Fig. S6 **a** shows the energy dependence of the above mentioned magnetic structures on the perpendicular external field $\mu_0 H_\perp$, which is opposite to the skyrmion polarity. The total energy of all three kinds of magnetic structures increases with the external field, and we observed the transformations of the skyrmionium (red line) and skyrmion bag (blue line) into skyrmion at $\mu_0 H_\perp$=14 mT and 18 mT respectively, as indicated by the sudden drop of their energy. In general, the topological charges of skyrmion bag have the highest energy cost, followed by the skyrmionium, and the skyrmion has the lowest energy cost. In our experiments, we sequentially observed the transformations of the net domain into skyrmion bag, skyrmionium and skyrmion. This process can be well explained by the relaxation process of an excited magnetic system in minimizing its total free energy.

The energy compositions of these magnetic structures are shown in Fig. S6 **b**. The negative energy components E_{dmi} and E_{demag} indicate that the magnetic structures are mainly stabilized by the DMI, while the magnetostatic effect also contributes. Moreover,

E_{dmi} of skyrmion bag is lower than that of skyrmion, which means DMI actually favors the formation of cluster-like spin textures. However, the clustering of isolated skyrmions leads to a significant increase in magnetic anisotropy energy E_{anis}, which forbids the formation of skyrmion bags with high topological charges. These results indicates that the skyrmion bags and skyrmionium may be more easily found in magnetic systems with low magnetic anisotropy, while have a strong antisymmetric exchange, and high saturation magnetization.

REFERENCES

1. Zhang, X. *et al.* Control and manipulation of a magnetic skyrmionium in nanostructures. *Phys. Rev. B* **94**, 094420 (2016).

2. Rybakov, F. N., and Nikolai S. K. Chiral magnetic skyrmions with arbitrary topological charge. *Phys Rev. B* **99**, 064437 (2019).

3. Leonov, A. O., and Catherine, P. Skyrmion clusters and conical droplets in bulk helimagnets with cubic anisotropy. *Phys. Rev. B* **99**, 144410 (2019).

4. Kind, C., and David F. Magnetic skyrmion binning. *Phys. Rev. B* **103**, L100413 (2021).

5. Foster, D., et al. Two-dimensional skyrmion bags in liquid crystals and ferromagnets. *Nat. Phys.* **15**, 655-659 (2019).

6. Powalla, L., et al. Seeding and Emergence of Composite Skyrmions in a van der Waals Magnet. *Adv. Mater.*, 2208930 (2023).
