# Supplementary material for: Reversible conversion between skyrmions and skyrmioniums
Source: Nat Commun. 2023 Jun 9;14:3406. doi: 10.1038/s41467-023-39007-1 (PMC10256730; doi:10.1038/s41467-023-39007-1)
Supplement: Supplementary file 3 — Description of Additional Supplementary Files [file 41467_2023_39007_MOESM3_ESM.docx]

**Inventory of Supporting Information**

1. Supplementary Video 1: The skyrmion is created in the device by a single pulse.
2. Supplementary Video 2: The creation process of the net domain. The sinusoidal pulses are injected into the Hall bar along +*x*.
3. Supplementary Video 3: The fragmentation process from the stripe domains to the isolated skyrmionium.
4. Supplementary Video 4: The conversion from a skyrmion bag to a skyrmionium. Square pulses are used to drive the skyrmionium and skyrmion into motion.
5. Supplementary Video 5: The conversion from a skyrmionium to a skyrmion.
6. Supplementary Video 6: The skyrmion exhibits SkHE motion.
7. Supplementary Video 7: The SkHE free motion of skyrmionium.
8. Supplementary Video 8: The role of different waveforms.
9. Supplementary Video 9: The creation of skyrmioniums with different sizes.
